# PA-Tran: Learning to Estimate 3D Hand Pose with Partial Annotation

**DOI:** 10.3390/s23031555

**Published:** 2023-01-31

**Authors:** Tianze Yu, Luke Bidulka, Martin J. McKeown, Z. Jane Wang

**Affiliations:** 1 Department of Electrical and Computer Engineering, University of British Columbia, Vancouver, BC V6T 1Z4, Canada; 2 Faculty of Medicine, University of British Columbia, Vancouver, BC V6T 1Z4, Canada

**Keywords:** 3D hand pose estimation, single RGB image, partial annotation, transformer, synthetic dataset, PD (Parkinson’s disease) hand dataset

## Abstract

This paper tackles a novel and challenging problem—3D hand pose estimation (HPE) from a single RGB image using partial annotation. Most HPE methods ignore the fact that the keypoints could be partially visible (e.g., under occlusions). In contrast, we propose a deep-learning framework, PA-Tran, that jointly estimates the keypoints status and 3D hand pose from a single RGB image with two dependent branches. The regression branch consists of a Transformer encoder which is trained to predict a set of target keypoints, given an input set of status, position, and visual features embedding from a convolutional neural network (CNN); the classification branch adopts a CNN for estimating the keypoints status. One key idea of PA-Tran is a selective mask training (SMT) objective that uses a binary encoding scheme to represent the status of the keypoints as observed or unobserved during training. In addition, by explicitly encoding the label status (observed/unobserved), the proposed PA-Tran can efficiently handle the condition when only partial annotation is available. Investigating the annotation percentage ranging from 50–100%, we show that training with partial annotation is more efficient (e.g., achieving the best 6.0 PA-MPJPE when using about 85% annotations). Moreover, we provide two new datasets. APDM-Hand, is for synthetic hands with APDM sensor accessories, which is designed for a specific hand task. PD-APDM-Hand, is a real hand dataset collected from Parkinson’s Disease (PD) patients with partial annotation. The proposed PA-Tran can achieve higher estimation accuracy when evaluated on both proposed datasets and a more general hand dataset.

## 1. Introduction

Given a video sequence or an RGB image captured from a camera or mobile device, the task of markerless pose estimation is to predict the positions of the body keypoints (including joints and vertices) relative to a certain coordinate system [1]. As one most frequently used parts that human beings interact with the environment, hand pose estimation (HPE) is of great research interest and has numerous applications in areas such as robotics, virtual reality (VR) and augmented reality (AR), AI-aided diagnosis and smart human-computer interaction (HCI) systems [2,3]. Apart from those downstream applications, HPE also plays an important role in many basic upstream tasks, including gesture recognition [4,5,6] and sign language recognition (SLR) [7,8]. Accurate hand pose estimation and reconstruction can significantly enhance the understanding of the learning and inference of human behavior, thus enabling a more intelligent interaction between humans and the target system with improved user experience.

In recent years, with the rapid development of hardware (e.g., Microsoft Kinect [9], Oak-D camera [10], wearable sensors) and advances in deep learning algorithms, HPE research has achieved considerable progress. The state-of-the-art approaches have achieved promising performance in a controlled environment with different data modalities, such as 2D images, 2D images with depth map [11,12,13], wearable gloves and sensors [3,14]. Among these modalities, since single-view 2D RGB images are much more available than sensors and depth images, HPE from a single RGB image can be widely used and easily deployed to various end devices. Meanwhile, HPE from a single RGB image is also a more challenging task in practice due to the following concerns:

1. Full Accurate Annotation: Creating a fully-annotated HPE dataset is time-consuming and requires sufficient human and financial resources. Meanwhile, the hardware for capturing the data, like multi-view and high-resolution cameras, is also costly.
2. Hand Occlusion: During the motion of performing a hand gesture, or holding some objects, the fingers of the same hand may cross over each other or be covered by other objects, making several keypoints unobservable. In such cases, certain hidden keypoint positions cannot be predicted only based on vision.
3. Low Resolution: In a practical scenario, the hand may only occupy a small area in the image, resolving a quite low hand resolution. For instance, even with a 4K capturing system, if the principal focus is not the hand and the object size is small due to the viewing distance, the hand may only occupy tens of pixels.
4. Motion blur: Due to the relatively low sampling rate of cameras in many practical scenarios (e.g., normally 15 fps or 30 fps), fast movements of the fingers will cause motion blur in the captured images and video sequences. Motion blur in the images could significantly affect both the hand pose annotation and estimation tasks.

To our best knowledge, no work in the literature has jointly addressed the above concerns, especially the partial annotation challenge. In this work, to fill this research gap, we propose a 3D hand pose estimation approach from a single RGB image with partial annotation. In summary, our contributions are as follows:

1. As the first attempt in the literature, we consider the partial annotation challenge in image-based 3D HPE and propose a novel partial annotation learning framework, PA-Tran, for 3D hand pose estimation.
2. Due to the lack of the required dataset in the literature, we created two hand datasets. The first (APDM-Hand) is a synthetic dataset for a specific task (with APDM accessories on the palm). The second is a real-hand dataset with partial annotation collected from PD patients wearing APDM accessories.
3. By explicitly encoding and exploring the label status (observed/unobserved) in the proposed PA-Tran, training with partial annotation is shown to be even more efficient than full annotation. We compare the performances of using different annotation percentages ranging from 50% to full annotation.

The rest of this paper is organized as follows. Section 2 introduces the related work. Section 3 presents the major components of the proposed model PA-Tran. Section 4 provides an experimental evaluation of the proposed model. Finally, Section 5 concludes the paper and provides potential directions for future research.

## 2. Related Work

**Figure 1:**
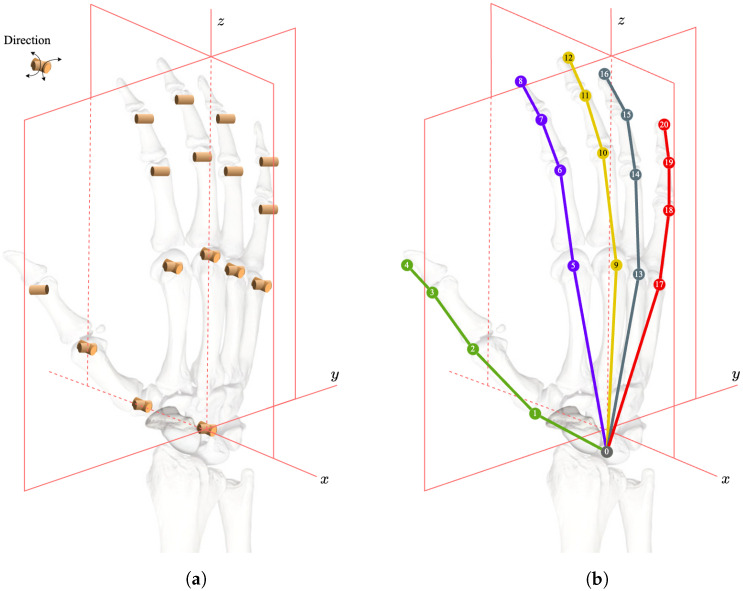
Biological characteristics of the human hand skeleton: (**a**) Illustration of the DoF of the hand; (**b**) Indices of the hand joints.

HPE is a long-standing research area due to its wide range of applications. The structure of the human hand is quite complex, with a lot of degrees of freedom (DOF). However, the biological structure enforces the motion of the hand to follow a specific pattern, as shown in Figure 1. The cylinder and bicylinder represent the flexibility of hand joints. This biological limitation also makes it possible to learn high-dimensional features from a 2D or 2.5D image. In recent years, with the increasing popularity of deep neural networks, researchers have proposed many methods to estimate the hand pose from images [15]. From the task requirement perspective, single-image-based HPE could be categorized into 2D estimation and 3D estimation, and 2D hand pose estimation is often referred to as hand keypoint detection.

*2D Hand Pose Estimation.* For the 2D hand keypoint detection task, Ref. [16] directly regresses the Cartesian coordinates of the keypoints using a normal convolution architecture.

After that, more works turn to regress images into confidence maps. Convolutional Pose Machine (CPM) [17] inputs the regressed confidence maps into convolutional architectures to learn implicit spatial dependencies. Ref. [18] designs a novel ’stacked hourglass’ architecture to predict 2D human pose by capturing and consolidating information across all image scales. In [19], hand pose estimation is separated into five independent finger pose estimations. Meanwhile, multi-view 2D keypoints could be combined to estimate the 3D pose. Ref. [20] presents a framework called multi-view bootstrapping that uses multi-view images to train keypoints detectors iteratively to denoise the prediction. Ref. [21] also utilizes the multi-view information and proposes an end-to-end single-stage convolutional neural network to estimate the coordinates of the hand keypoints. The multi-view approach can achieve good performance in an experimental environment. Nevertheless, it is not always feasible to be deployed to a practical environment for two main reasons: first, the multi-view camera system may not always be available; and multi-view feature fusion has high requirements for the consistency and synchronicity of different data acquisition channels.

*3D Hand Pose Estimation.* Compared with the multi-view-based approach for 3D HPE, the single-RGB-image condition has lower requirements for hardware devices and less restriction on implementation scenarios [22]. One commonly used approach is extracting the image features using convolutional neural networks (CNNs). Different from previous work, in [23], the researchers, for the first time, propose a learning-based architecture to estimate 3D hand pose from a single RGB image. They use synthetic data with various augmentation options and sequently design the three networks, i.e., HandSegNet, PoseNet, and PoserPrior, showing the possibility of predicting reasonable 3D hand poses from 2D keypoints. As a new type of convolutional neural network, the graph convolutional neural network (GCN) has been used to estimate the relationship in knowledge graphs and, more recently, in many areas of computer vision. Integrating GCN for RGB image-based hand pose estimation has become a new direction. Similarly to [23], a framework based on graph convolutional neural networks, HOPE-Net, is proposed in [24]. HOPE-Net uses a cascade of two adaptive graph convolutional neural networks. One network estimates the 2D coordinates of the hand joints and the object’s corners. The 2D coordinates predictions are passed to the second adaptive Graph U-Net to estimate the 3D coordinates of both the hand and the object. Compared with the works above, some methods have changed to estimate the 3D coordinates directly. Ref. [25] proposes a weakly-supervised method that could be trained without using any paired 2D-to-3D supervision. Ref. [26] presents the first end-to-end deep learning method for 3D hand pose estimation from RGB images in the wild and utilizes a 3D to 2D reprojection loss. Ref. [27] propose the first large-scale multi-view hand dataset FreiHAND with 3D hand pose and shape annotations. And a framework aggregating information from all the multi-view cameras is proposed to predict a single 3D hand pose. I2L-MeshNet [28] is a novel network for 3D pose and mesh estimation from a single RGB image. It consists of two modules: PoseNet and MeshNet. PoseNet estimates the three lixel(line+pixel)-based 1D heatmaps of all joints. MeshNet takes the pre-computed image features from the PoseNet and estimates the hand shape. Ref. [29] proposes a novel framework based on the vision transformer and achieves state-of-the-art results. In the meantime, hand pose estimation also started to be explored in more directions and scenarios. Ref. [30] proposes a multi-modal approach that uses 2D labels on RGB images as weak supervision to perform 3D HPE. And the multi-modal architecture also incorporates the camera and LiDAR with an auxiliary segmentation branch. Ref. [31] proposes a new scenario when the input images come from a single fisheye camera.

*Partial Annotation Learning.* As a non-fully-supervised direction (e.g., semi- supervised and weakly-supervised), learning with partial annotation has been actively studied very recently in many topics, including multi-label image classification [32], object detection [33], and segmentation [34]. Hand pose estimation is inherently a partial annotation learning problem as, most of the time, only a part of the hand is visible in an image because of the high DOFs of the hand. However, only a few works with semi-/weakly-supervision have been studied for hand pose estimation. Ref. [35] proposes a weakly-supervised network for training with RGB images and corresponding depth maps, and does inference with RGB images only. In [36], the researchers propose a semi-supervised framework to form a shared latent space between the synthetic depth image, real depth image, and pose. These works leverage the depth information to train the model in a weakly-supervised manner and don’t focus on partial annotation learning for hand pose estimation. While in our settings, the input is RGB images captured from general webcams and has no depth information. To fill this research gap, we propose a framework for single-image-based 3D hand pose estimation with partial annotation by jointly estimating the status and the position of hand keypoints. Table 1 provides a summary to illustrate different assumptions and problem settings of related hand pose estimation approaches in the literature.

*Synthetic Hand Dataset.* Acquiring full annotations for real images is complicated and may not be feasible in practice as it requires complex setups and labor-intensive manual annotations in different perspectives, Refs. [37,38] create synthetic datasets to help relieve the problem of lacking fully-annotated hand data. Within these synthetic datasets, Dart [38] explores the hand synthetic dataset to a new frontier that generates synthetic hand data with several accessories like watches and rings. However, as required by our specific motivating application in Parkinson’s disease research, we have a practical demand to estimate the hand pose with a wearable sensor on the palm, which will significantly affect hand pose estimation performance when applying pre-trained methods. Motivated by Dart [38], we generate a synthetic dataset called APDM-Hand, to help improve the performance of HPE in our specific scenario in the PD study. APDM-Hand contains hand images wearing an accessory of the APDM sensor on the palm and could be treated as an additional subset of Dart. The description of APDM-Hand is detailed in Section 4.

## 3. Method

### 3.1. Problem Setup

In the regular 3D hand pose estimation task, the goal is to predict a set of coordinates, including hand joints and vertices, from an input hand image. Let I denote an input image, and $y_{gt}\in R^{K\times 3}$ be the fully-annotated ground truth of the coordinates, where *K* is the number of the keypoints. Hand pose estimation is to construct a model f() to predict a set of coordinates given an input I, formulated as $\hat{y}=f\left(I\right)$. Conventional HPE methods take the image I as the input and train the model with fully-annotated $y_{gt}$, including both visible and invisible keypoints. However, HPE is inherently a partial-label learning problem, where the hand generally could be partially-visible in a practical scenario. With a subset of labels $y_{o}\subseteq y_{gt}$ being observed, in this reformulated partial-annotation setting, our goal is to train/predict the unobserved keypoints ${\hat{y}}_{u}=\hat{y}\setminus y_{o}$. Meanwhile, the hand in an image is always with low resolution and occlusion. It is infeasible to annotate all the keypoints accurately. Therefore, we propose a data-driven approach for 3D hand pose estimation with partial annotation, which is referred to as PA-Tran.

### 3.2. Pa-Tran

**Figure 2:**
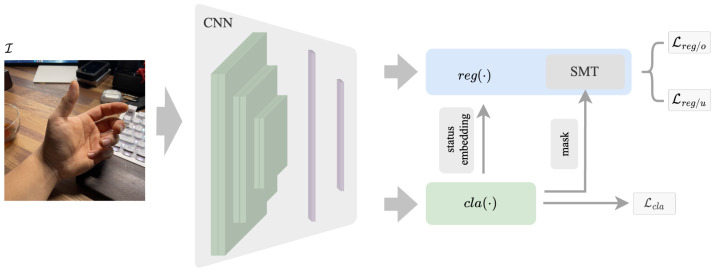
Overview of the proposed PA-Tran framework. Given an input image I, we extract the image features using a convolution neural network. Then the image features are passed into two separate branches: the regression branch reg(·) and the classification branch cla(·). cla(·) will generate the status embedding for reg(·) and masks for SMT to learn the interaction between labels. The structures of reg(·) and cla(·) are detailed in Section 3.2.

The main challenge in hand pose estimation with partial annotation is integrating the partial annotation information into a regression problem. To address this issue, we separate the estimation problem into two tasks and propose a dual-branch structure. The proposed PA-Tran framework is shown in Figure 2, which consists of the CNN feature extractor, the classification branch cla(·) and the regression branch reg(·). Compared with conventional ViT [39], using CNN as the feature extractor could effectively utilize the local receptive fields of CNN and global receptive fields of Transformer, especially for image-based tasks. The approach has been proven to be quite effective and can also be applied with pre-trained networks such as ResNet [40]. Besides, it can alleviate the demand for large-scale training datasets of Transformer to some extent. The classification branch is deployed to estimate the status of the keypoints, and the regression branch is used to estimate the positions of the keypoints. The two branches work dependently.

#### 3.2.1. Regression Branch

**Figure 3:**
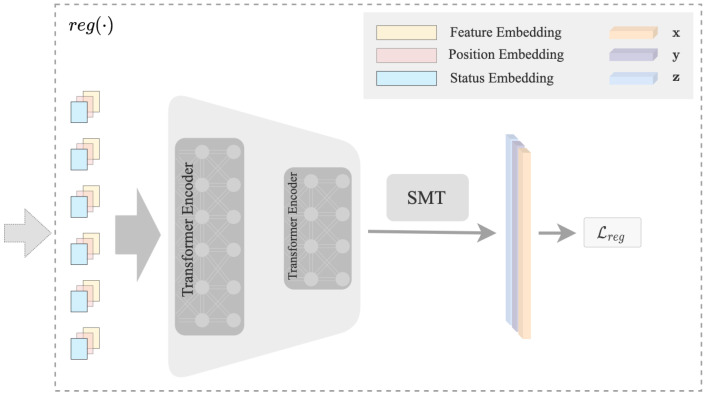
The structure of the reg(·) branch. The input is the concatenation of feature embedding, position embedding, and status embedding. Sequential transformer blocks are adopted to reduce the dimension of the hidden embedding progressively. The final output is the coordinates of the keypoints.

The structure of the regression branch reg(·) is shown in Figure 3. The input consists of three parts: feature embedding, position embedding, and status embedding. Inspired by the excellent performance of capturing the dependencies between different variables of ViT [39], we adopt Transformer to model the interactions among the three representation embeddings. In addition, Transformer encoders are order-invariant, allowing for any type of dependencies between all features and labels to be learned [41]. The proposed input format allows us to easily input the representation embeddings into the Transformer encoder. Inspired by [29], we apply a similar structure to reduce the dimensionality of the hidden embedding after each encoder layer progressively, and the final outputs of the encoder are the coordinates of the keypoints.

*Feature Embedding:* Following the structure in [42], we adopt a convolutional neural network based module for extracting features. Given an input image $I\in R^{H\times W\times 3}$ where *H* and *W* represents the height and width of I respectively, we extract the image feature vector $h\in R^{2048\times 1}$.

*Position Embedding:* Each keypoint whose coordinates $\left(x_{k},y_{k},z_{k}\right)$ are from a predefined hand template is concatenated to the feature embedding, which is equivalent to the position embedding in [39]. We also prove the equivalence as follows.

**Theorem 1.**  *Keypoint position concatenation is equivalent to position embedding.*

**Proof.**  We use ${\left[h,p\right]}^{T}\in R^{(D+N)\times 1}$ to represent the concatenation of the feature vector ($h\in R^{D\times 1}$) from the CNN backbone and the position embedding $p\in R^{N\times 1}$. For a linear transformation with the matrix $w\in R^{d_{t}\times \left(D+N\right)}$ where $d_{t}$ represents the target dimension, $Em_{*}$ represents the embedding, the operation could be formulated as

(1) $$w\cdot {\left[h,p\right]}^{T}=\left[w^{h},w^{p}\right]\cdot {\left[h,p\right]}^{T}=w^{h}\cdot h+w^{p}\cdot p=Em_{feature}+Em_{position}$$

□

*Status Embedding:* An additional status embedding, $s_{k}$’s, is added to the input of the Transformer encoder where *k* is the index of the corresponding keypoint. We consider the status (i.e., ‘*o*’ for observed and ‘*u*’ for unobserved) an extremely strong feature for querying. As shown in Figure 1a,b, the hand’s degrees of freedom are quite limited. And the keypoints sets {3, 7, 11, 15, 19} and {6, 10, 14, 18} could only move in one direction, and the set {1, 2, 5, 9, 13, 17} could move in two directions (the keypoints of fingertips {4, 8, 12, 16, 20} are excluded). In this way, if the status (observed or unobserved) of the keypoints is a priori knowledge, the approximate structure of the hand is constrained within a small range. For instance, if all keypoints are observed, the palm must be spread out to some extent. We use this observation as a constraint to help improve the accuracy of the proposed HPE task. In addition, it enables us to use partially labeled annotation by adding the status embedding to the input embeddings. We employ a data-driven approach for generating the status embedding, which is accomplished by the classification branch.

#### 3.2.2. Classification Branch

In the classification branch cla(·), we formulate the hand pose estimation problem as a partial multi-label (PML) image classification problem. Multi-label learning with partial annotation has recently been an active topic with practical importance since full annotations are generally hard to acquire for multi-label images. In this branch, we treat the keypoints as independent instances in a multi-label image and predict the status of each keypoint. The prediction will be used as the status embedding in the regression branch described above. Meanwhile, we also use the classification branch to simulate the procedure of annotating. By selecting the top n% predictions of cla(·) as annotations for the selective mask training(SMT), we further demonstrate that partially annotating the dataset could be more efficient in the ablation study.

#### 3.2.3. Model Training

*Loss functions:* Uniforming the annotation format of different datasets generally could be infeasible as each dataset has its own target task and annotation budget. Therefore, we take both the 3D and 2D annotations into consideration. Given a dataset D and the corresponding keypoints annotation (including both vertices and joints in 3D ($K_{3D}$) and 2D ($K_{2D}$), the loss function of the regression branch is defined as:

(2) $$L_{reg}=\alpha \left(L_{K_{3D}}+L_{K_{rep2D}}\right)+\beta L_{K_{2D}}$$

where the three components are the losses with 3D annotation, 2D annotation, and 3D to 2D projection annotation [43]. As data from different sources may have different annotation formats, not all datasets could provide both 2D and 3D annotations at the same time. Therefore, α,β∈{1,0} is used to represent the existence of the corresponding annotation format. The camera parameters π(·) is learnable by optimizing the target function

(3) $$L_{K_{rep2D}}=||\pi \left(K_{3D}\right)-K_{2D}\left|\right|$$

Follow the work in [28], we use the L1-loss to optimize $L_{reg}$. Considering the various annotation formats of different datasets, $K_{3D}$ and $K_{2D}$ could be optional simultaneously, but including more annotations with different formats will help improve the estimation accuracy.

We apply the binary cross-entropy (BCE) loss for the classification branch. Conventional partial multi-label image classification always has a data imbalance problem because the number of the total category is large. Still, the number of objects in a single image is quite limited (e.g., a few). While for a hand dataset, the imbalance issue is less of a concern in the proposed scenario, as the situation where the number of one keypoint is tens of times more than another one hardly exists. Therefore, it is reasonable to apply the BCE loss, and thus the loss of the classification branch $L_{cla}$ is defined as:

(4) $$L_{cla}=-\frac{1}{K}\sum_{i=1}^{K}\left(y^{i}\log\left({\hat{y}}^{i}\right)+\left(1-y^{i}\right)\log\left(1-\left({\hat{y}}^{i}\right)\right)\right)$$

*Selective Mask Training (SMT):* The status embedding enables us to integrate the prior knowledge of whether the corresponding keypoints show up in the PA-Tran model. Inspired by the previous works [44], which employ the masked language model training to predict missing words from the context, we adopt a similar strategy called Selective Mask Training (SMT). In masked language modeling, the length of the input query varies, and the missing words are also random, so the masks for training will be generated randomly. However, different from the language model that masks the input query at random, the number of the keypoints is fixed in HPE, and the presence or absence of hand keypoints follows a specific pattern that is learnable from the label status embedding. Therefore, we use the predicted keypoints status to guide the masks in HPE, and the process is referred to as Selective Mask Training. By masking a specific amount of labels of unobserved queries, the potential combinations of the keypoints status will be learned. Meanwhile, our model will predict all keypoints, including both observed and unobserved ones. Therefore, the overall loss function is defined as:

(5) $$L_{\Sigma }=L_{cla}+\left(L_{reg/o}+\gamma L_{reg/u}\right)$$

where $L_{reg/o}$ and $L_{reg/u}$ are the losses of observed and unobserved keypoints and γ is a hyper parameter balancing training goals. In order to predict the value of a masked/unobserved query, SMT enforces the model to learn and utilize the inner connection with other related queries. In summary, the learning target of PA-Tran is separated into two: retrieve the values of the observed queries ($L_{reg/o}$) and retrieve the values corresponding to the unobserved queries with observed queries ($L_{reg/u}$).

## 4. Experiments

For the experiments, we first compare the performance of the proposed PA-Tran for single-RGB-image-based 3D hand pose estimation with several popular approaches on two datasets. Then in the ablation study, we evaluate the proposed PA-Tran from different aspects to demonstrate its effectiveness when using partial annotation. We also show the qualitative results of the proposed PD-APDM-Hand dataset.

### 4.1. Dataset and Setup

FreiHAND [27] is a real image dataset captured using multi-view devices. They captured hand gestures from 32 subjects of different genders and ethnic backgrounds. The dataset consists of 134 k images (130 k for training and 4 k for evaluation), with a resolution of 224×224. A detailed and accurate annotation in different formats is also available for each image, including 2D/3D annotation, mesh annotation, masks, and camera parameter matrices. Due to task-related purposes, we only consider the scenario that there are no extra objects in the hand. So we intentionally exclude most of the images that hold objects to put more emphasis on estimating the hand itself.

**Figure 4:**
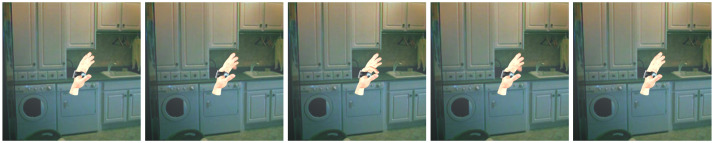
Examples of finger-tapping animation frames with motion blur.

**Figure 5:**
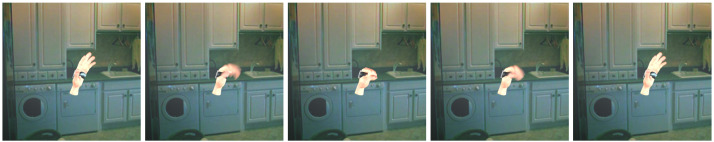
Examples of hand-movement animation frames with motion blur.

**Figure 6:**
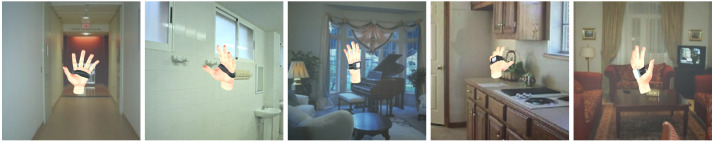
Examples of APDM-Hand images from different views and backgrounds.

APDM-Hand is a synthetic dataset. In this work, we propose a synthetic hand dataset generated with Blender [45] and Unity [46]. Unlike previous synthetic datasets, our APDM-Hand is designed specifically for hand pose estimation with APDM sensors [47] on the palm, as using an APDM sensor on the palm is a typical experimental setting when simultaneously collecting both video and motion sensor data during hand movement tasks. While directly applying HPE methods (which are trained on no-sensor hand images) to such images will result in poor or even odd estimation results. Compared with other synthetic hand datasets, as shown in Table 2, APDM-Hand extends the current datasets from the on-hand accessory aspect. In addition, APDM-Hand also includes image sequences (i.e., video) and motion blur simulations, which other datasets don’t include. Figure 4, Figure 5 and Figure 6 show several images and video sequence examples under different conditions.

More specifically, our APDM-Hand dataset has the following features.

1. Hand task classes: The two hand task classes in our dataset are based on two popular actions performed for the Unified Parkinson’s Disease Rating Scale (UPDRS) test in PD studies [51]. An animation video was created for each action: ‘finger tapping’ and ‘hand motion’. The former is a rapid tapping of the index finger and the thumb, and the latter is a full clench of the fist followed by a full extension such that the hand is fully opened. The ‘finger tapping’ animation is 41 frames in length, and the ’hand motion’ animation is 21 frames in length.
2. Backgrounds: The dataset draws from a pool of 50 backgrounds taken from a public indoor scene dataset [52]. The particular backgrounds were chosen for their day-to-day relevance, consisting of bathrooms, bedrooms, hallways, home offices, kitchens, living rooms, offices, pools, restaurants, transit stations, storage spaces, stores, and studios.
3. Varied views: There are 25 camera views in the dataset. 12 views face the front of the hand, and 13 views face the back of the hand. For each view, 25 backgrounds are uniformly sampled from the 50 total backgrounds, and each selected background is used for both ‘finger tapping’ and ‘hand motion’ animations. This results in a total of 1550 frames per view.
4. Motion blur: The dataset applies different camera settings (e.g., sampling rates, shutter speed) to simulate real scenarios when motion blur happens.

**Figure 7:**
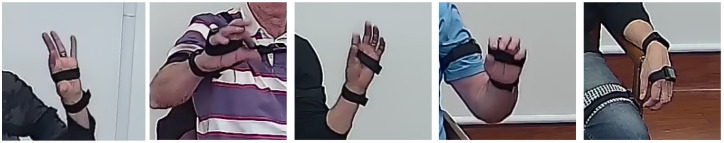
Examples of PD-APDM-Hand, which is collected from real Parkinson’s Disease patients when taking the UPDRS test.

PD-APDM-Hand is a real video dataset with partial annotation, and example images are shown in Figure 7. The videos are collected from 9 Parkinson’s Disease patients and 2 Healthy Control (HC) subjects with APDM wearable sensors on their hands and partially annotated with 2D annotations. Original videos are collected with 4k@15fps when the subjects perform finger tapping and hand movement. There are 4392 images extracted from the videos, and the hands are resized to 224×224. We fine-tuned our model on the PD-APDM-Hand dataset and reported qualitative results.

### 4.2. Evaluation Metrics

To fairly evaluate different methods, we report the HPE results using standard performance metrics: Mean-Per-Joint-Position-Error(MPJPE) [53] and Mean-Per-Vertex-Position-Error(MPVPE) [54]. MPJPE is a metric for evaluating 3D pose. MPJPE measures the Euclidean distances between the predicted joints and the ground truth points. PA-MPJPE, or Reconstruction Error, is another metric for this task. It first performs a 3D alignment using Procrustes analysis (PA) and then computes MPJPE. PA-MPJPE is commonly used for evaluating 3D reconstruction as it measures the errors of the re-constructed structure without regard to the scale and rigid pose (i.e., translations and rotations). Similar to MPJPE, MPVPE measures the Euclidean distances between the ground truth and predicted vertices.

### 4.3. Implementation Details and Setup

For the convolutional neural network, we apply HRNet [55] pretrained on ImageNet [56] as the backbone. The dimension of the output feature is 2048×1. For reg(·), PA-Tran uses four attention heads and four layers for each transformer block. For cla(·), the data from the CNN backbone are passed to a two-layer fully connected layer, then using the sigmoid function for multi-label classification. Adam [57] is used as the optimizer. The learning rate starts with $10^{-4}$ and a decay factor of 10 after every 80 epochs. We compare the evaluation metrics of our proposed method with the representative and state-of-the-art 3D pose and mesh estimation methods. HMR [25] is the first end-to-end adversarial method to recover human pose and shape without using 2D to 3D supervision. Ref. [26] is a novel end-to-end representation learning method for 3D hand pose estimation from RGB images in the wild. MVNet [27] is the baseline method of the FreiHAND dataset. I2LMeshNet [28] achieves the top result on the challenge of estimating the 3D pose in the wild. METRO [29] is the state-of-the-art method that is able to be configured to the proposed scenario for our task purposes. All methods use the same percentage of annotations for the partial annotation scenario. As the compared methods are not designed originally for partial annotation learning, the loss of missing annotations will be set to zero directly when calculating the loss function, which follows a typical setting of partial annotation learning in multi-label image classification.

### 4.4. Results

**Figure 8:**
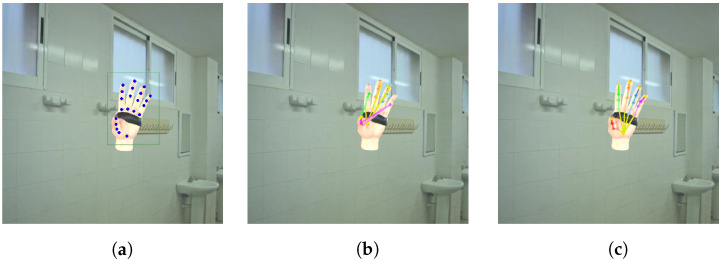
Qualititive result on APDM-Hand dataset: (**a**) Ground truth; (**b**) METRO; (**c**) PA-Tran.

First, we compare the results on the general real image dataset FreiHAND, and the HPE estimation results are shown in Table 3. The proposed PA-Tran outperforms the comparison methods in all evaluation metrics. Among these, the improvement of the prediction of joints is larger than vertices. In addition, we evaluate the performance of the methods on the proposed synthetic dataset APDM-Hand, and the results are shown in Table 4 and Figure 8. The proposed method also achieves the best performances on the APDM-Hand dataset. It should be noted that different datasets may have different hand sizes and environment settings, so the results are only comparable within the same dataset. The image sources of the two datasets introduce different challenges to the model. FreiHAND dataset captures real images; hence the data distribution and hand texture are closer to a real scenario. For APDM-Hand, although we have added more details like texture and light sources, the domain gap between real and synthetic data still exists.

Second, by exploring partial annotation of different percentages, we demonstrate that annotating partially could be more efficient. As shown in Table 5, we observe that training with full annotation cannot achieve the best performance, and there is nearly no improvement in increasing the annotation portion from 90% to full annotation. The best performance is achieved when using about 85% annotations. Actually, 80% to 85% annotation is a reasonable range in practical scenarios. One potential reason for this observation is the following: increasing the annotation percentage will increase the accuracy of the classification branch. However, an over-accurate estimation of the keypoints status can weaken the generalization ability of the regression model. Besides, introducing more annotations without representation features may cause performance degradation in the final accuracy. It is worth emphasizing that, as illustrated in Table 6 (85% vs. 100%), similar observations are also noted in other methods.

**Figure 9:**
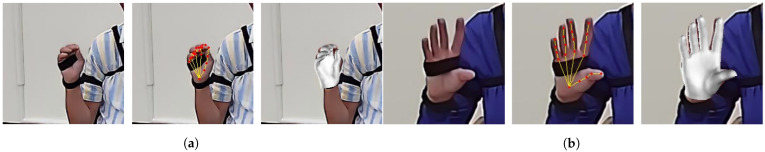
Qualititive results on PD-APDM-Hand dataset: (**a**) PD subject 1; (**b**) PD subject 2.

**Figure 10:**
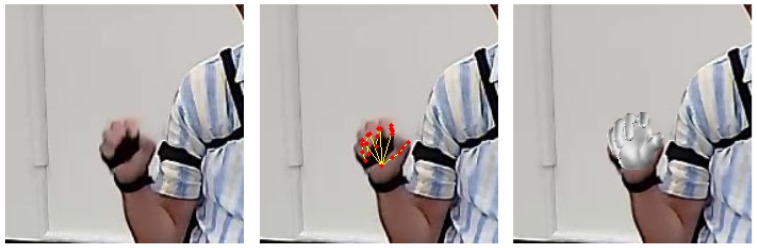
Hand pose estimation with motion blur.

Third, for the task of estimating the hand pose of PD patients, we performed an experiment on the proposed PD-APDM-Hand dataset. The model is pre-trained with FreiHAND and APDM-Hand, and fine-tuned with the PD-AMDM-hand. Some qualitative results are shown in Figure 9. We could see that the proposed method can accurately estimate the keypoints (joints and vertices). In addition, we also tested the proposed method when motion blur happens. Some qualitative results are shown in Figure 10. We could observe that even when the hand is blurry, the proposed method could estimate the hand pose accurately to some extent.

In summary, to assess the effectiveness of the proposed method and dataset, we explore several situations. According to the experiment results, simply increasing the number of annotations does not lead to an increase in accuracy. A potential cause for the result is analyzed above. Besides, more details need to be considered when generating synthetic data to reduce or even eliminate the domain gap between synthetic data and real data. The qualitative result on the real PD dataset also demonstrates the robustness of the proposed method. Nevertheless, the proposed method also has its limitations. First, due to our task of interest, we do not consider the situation of holding objects in the hand. Second, there still exists the domain gap between the proposed synthetic dataset and a real hand dataset, as discussed later in the ablation study.

### 4.5. Ablation Study

We first conduct an ablation study with/without the status embedding on the FreiHAND dataset. Secondly, we conduct experiments with/without SMT. The HPE results are aggregated in Table 7. The accuracy is much improved with the status embedding and selective mask training components.

Meanwhile, we also run an experiment under the cross-dataset setting, which trains the model on one dataset and tests it on another. The results are shown in Table 8, and we could acquire some useful information from the result. First, enlarging the training dataset by combining the two datasets could improve the performance of each dataset. Second, there is a strong domain gap between the two datasets as the accuracy of cross-dataset evaluation drops significantly.

## 5. Conclusions

We propose the novel PA-Tran framework for single-image-based 3D hand pose estimation with partial annotation. By jointly estimating the keypoints status and hand pose, the proposed PA-Tran could efficiently leverage the partial annotation. Meanwhile, with the introduction of the selective mask training mechanism, PA-Tran is able to learn the interaction between observed queries and unobserved queries. Experiments show that the proposed PA-Tran could achieve the best performances on three different datasets. Due to the lack of required datasets in the literature, we propose two hand pose datasets: one synthetic hand dataset and one real hand dataset captured from Parkinson’s disease patients. In the future, by exploring partial annotation, we plan to extend the proposed method to other tasks (e.g., body pose estimation, facial expression recognition) and downstream applications such as AI-aided auto-diagnosis of Parkinson’s Disease. Meanwhile, the proposed synthetic dataset will be improved to cover more general scenarios. To facilitate the study on hand pose estimation for specific applications (PD diagnosis), we would allow access to the datasets for research purposes.

## Figures and Tables

**Table 1:** Summary of problem settings in related 3D hand pose estimation methods.

Literature	Training/Testing Data	Network	Supervision
Tomos et al. [20]	multiview RGB/RGB	CNN	fully-supervised
Li et al. [21]	multiview RGB/RGB	CNN	fully-supervised
Zihao et al. [13]	RGB-D/RGB-D	-	-
Gyeongsik et al. [28]	RGB/RGB	CNN	fully-supervised
Christian et al. [23]	RGB/RGB	CNN	fully-supervised
Lin et al. [29]	RGB/RGB	Transformer	fully-supervised
Bardia et al. [24]	RGB/RGB	CNN, GCN	fully-supervised
Zhaohui et al. [12]	Depth/Depth	CNN	fully-supervised
Kanazawa et al. [25]	RGB/RGB	GAN	weakly-supervised
Yujun et al. [35]	RGB, 2D heatmap/RGB	CNN	weakly-supervised
Abdi et al. [36]	Depth/Depth	VAE-GAN	semi-supervised
Ours	RGB/RGB	CNN, Transformer	semi-supervised

**Table 2:** Comparison between different synthetic datasets.

	RHD [48]	GANH [49]	ObMan [50]	DART [38]	APDM-Hand
Accessories	✗	✗	✗	✓	✓
Palm accessories	✗	✗	✗	✗	✓
Video	✗	✗	✗	✓	✓
Motion blur	✗	✗	✗	✗	✓
2D/3D annotation	✓	✓	✓	✓	✓
mesh annotation	✗	✗	✓	✓	✓
mask	✓	✗	✓	✗	✓

**Table 3:** Comparisons of the 3D HPE results with SOTA methods on FeriHAND dataset.

Method	PA-MPJPE	PA-MPVPE
HMR [25]	−	13.2
Boukhayma et al. [26]	−	13.0
MVNet [27]	−	10.7
I2LMeshNet [28]	7.2	7.3
METRO [29]	6.3	6.4
PA-Tran	6.0	6.3

**Table 4:** Comparisons of the 3D HPE results with SOTA methods on APDM-Hand dataset.

Method	PA-MPJPE	PA-MPVPE
MVNet [27]	28.9	23.3
I2LMeshNet [28]	22.8	19.8
METRO [29]	16.1	13.4
PA-Tran	11.8	10.1

**Table 5:** Experiments of controlling the partial annotation percentage from 50% to full annotation.

Annotation Percentage	50%	60%	70%	80%	85%	90%	100%
PA-MPJPE	11.3	8.9	6.5	6.1	6.0	6.2	6.2

**Table 6:** PA-MPJPE with the annotation percentage of 85% and 100%.

Annotation Percentage	85%	100%
I2LMeshNet	7.2	7.4
METRO	6.3	6.5

**Table 7:** FreiHAND dataset: Accuracy results w/o status embedding and SMT.

	PA-MPJPE	PA-MPVPE
w/o $s_{k}$	6.8	6.9
w/o SMT	6.6	7.1
w $s_{k}+SMT$	6.0	6.3

**Table 8:** 3D HPE results in terms of PA-MPJPE under the cross-dataset setting.

	Train	FreiHAND	APDM-Hand	Combined
Test				
FreiHAND		6.0	23.3	5.9
APDM-Hand		15.6	11.8	11.2

## Data Availability

The data used in this study are available on request from the corresponding author.
